# Supplementary material for: Cryptosporidium uses CSpV1 to activate host type I interferon and attenuate antiparasitic defenses
Source: Nat Commun. 2023 Mar 16;14:1456. doi: 10.1038/s41467-023-37129-0 (PMC10020566; doi:10.1038/s41467-023-37129-0)

Supplementary Information

Cryptosporidium uses CSpV1 to activate host type I interferon and attenuate antiparasitic defense

Silu Deng[1,2], Wei He[2], Ai-Yu Gong[1,2], Min Li[2], Yang Wang[2], Zijie Xia[2], Xin-Tiang Zhang[2], Andrew S. Huang Pacheco[3], Ankur Naqib[4], Mark Jenkins[5], Patrick C. Swanson[2], Kristen M. Drescher[2], Juliane K. Strauss-Soukup[6], Michael Belshan[2], and Xian-Ming Chen[1,2]**

[1]Department of Microbial Pathogens and Immunity, Rush University Medical Center, Chicago, Illinois, USA; [2]Department of Medical Microbiology and Immunology, Creighton University School of Medicine, Omaha, Nebraska, USA; [3]Pediatric Gastroenterology, Children's Hospital & Medical Center, University of Nebraska Medical Center, Omaha, Nebraska, USA; [4]Department of Anatomy and Cell Biology, Rush University Medical Center, Chicago, Illinois, USA; [5]Animal Parasitic Diseases Laboratory, Agricultural Research Service, the United States Department of Agriculture, Beltsville, Maryland, USA; [6]Department of Chemistry and Biochemistry, Creighton University College of Arts and Sciences, Omaha, Nebraska, USA.

*****Correspondence and requests for materials should be addressed to X-M. Chen: xian_m_chen@rush.edu

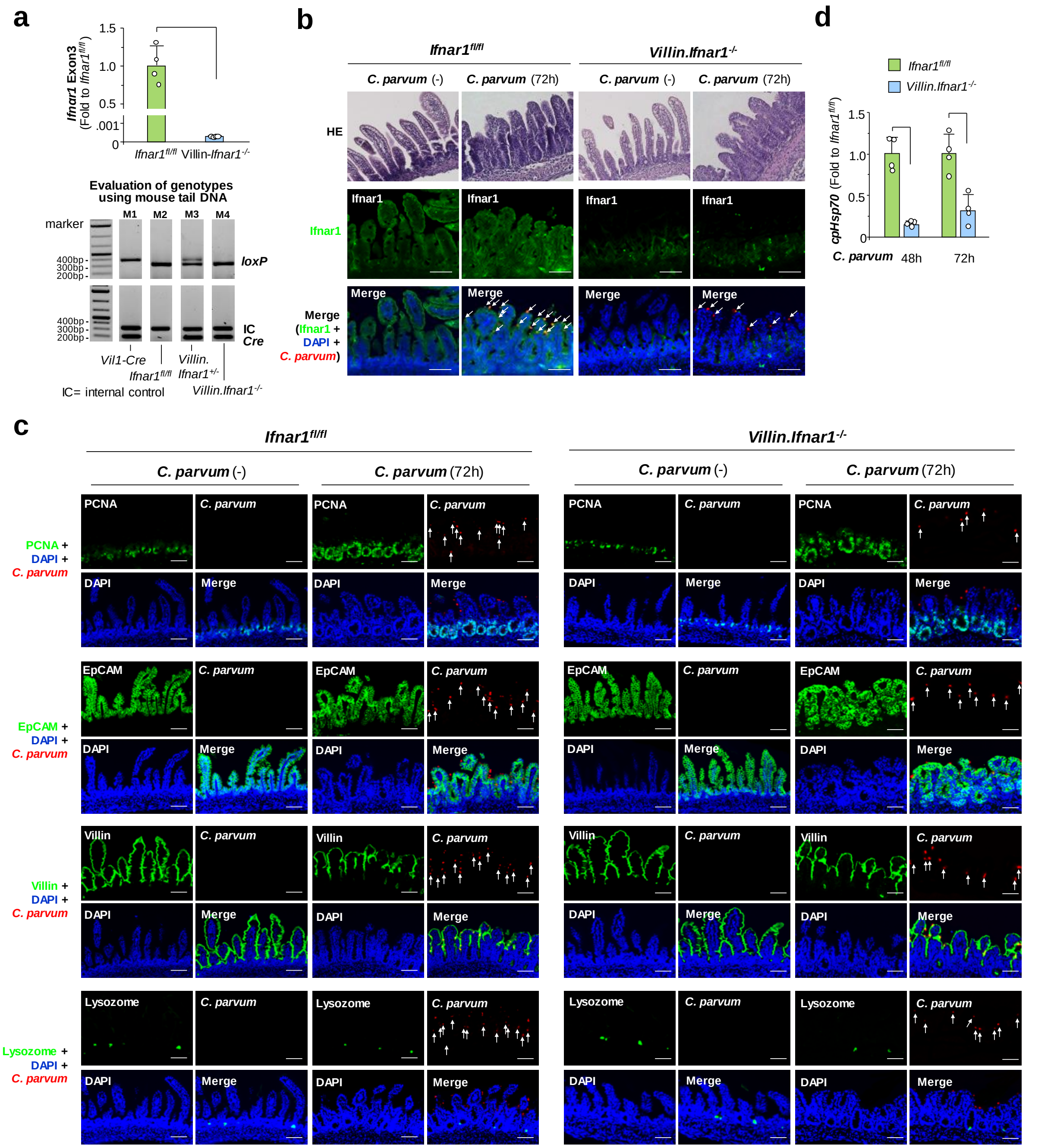

Supplementary Figure 1. Intestinal characteristics of *Ifnar1*$^{fl/fl}$ and *Villin.Ifnar1*$^{-/-}$ neonates following *C. parvum* infection.
a, Conditional intestinal epithelial type I IFN receptor *Ifnar1* knockout mice (*Villin.Ifnar1*$^{-/-}$ mice) from the cross-breeding of the *Ifnar1*$^{fl/fl}$ mice and the Vil1-cre mice. **b**, Ileal epithelial HE morphological features and Ifnar1 staining of *Ifnar1*$^{fl/fl}$ and *Villin.Ifnar1*$^{-/-}$ neonates following *C. parvum* infection. Neonates of 5 days old were orally administrated *C. parvum* oocysts (10^5/per neonate) and ileal tissues (2 cm of small intestine tissue from the ileocecal junction) were collected at 72h p.i., followed by HE staining and immunostaining for Ifnar1 and *C. parvum*. Neonates received PBS were used as the control. **c**, Ileal epithelial labeling of EpCAM and Villin and distribution of Paneth cells and proliferating cells in the WT control littermates (*Ifnar1*$^{fl/fl}$) and *Villin.Ifnar1*$^{-/-}$ neonates following *C. parvum* infection (72h p.i.). Ileal tissues were collected followed by immunostaining for PCNA (positive staining of proliferating cells), EpCAM, Villin, and lysozome (marker for Paneth cells). Representative images from 4 independent experiments are shown (in **b** and **c**); Blue: DAPI (DNA), green: Ifngr1 (in b) or PCNA or EpCAM or Villin or Lysozome (in c), red: *C. parvum* (arrows). Bars = 50 µm. **d**, *C. parvum* infection in intestinal epithelium from *Ifnar1*$^{fl/fl}$ and *Villin.Ifnar1*$^{-/-}$ mice at 48h and 72h p.i. Infection burden was evaluated by RT-qPCR of *C. parvum Hsp70* (*cpHsp70*) and presented as fold changes to *Ifnar1*$^{fl/fl}$ normalized to host *Gapdh*. Data are presented as mean values ± SD, compiled from 4 independent experiments and the dots represent the mean value of each experiment with 6 mice in each group (in **a** and **d**). *p* values were determined by two-tailed unpaired Student's *t*-test. Source data are provided as a Source Data file.

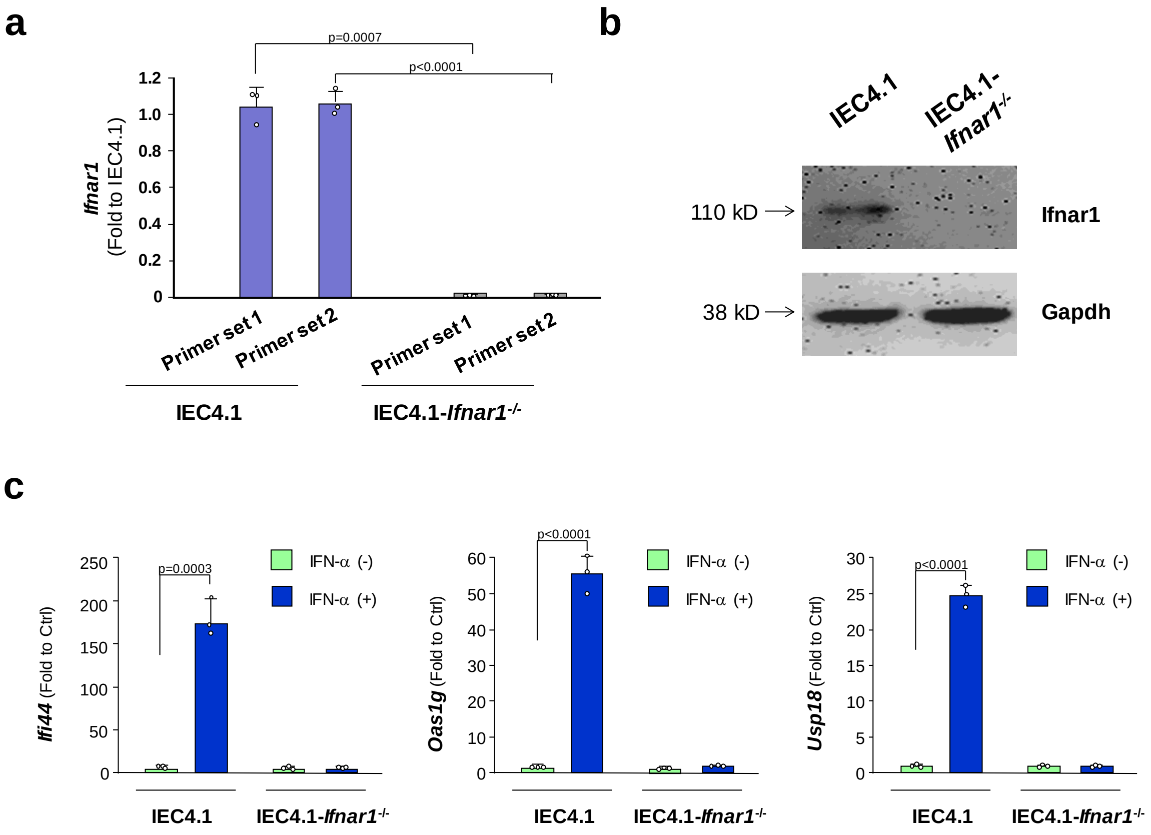

Supplementary Figure 2. Generation of stable IEC4.1 cells deficient in *Ifnar1* using the CRISPR/Cas9 approach.

a and **b**, Stable IEC4.1 cells deficient in *Ifnar1* (IEC4.1-*Ifnar1*$^{-/-}$ cells) were generated through transfection of IEC4.1 cells with the *Ifnar1*-CRISPR/Cas9 KO and the *Ifnar1*-HDR plasmid. Deletion was verified by RT-qPCR using two separate PCR primers covering the designed regions of *Ifnar1* (**a**) and by Western blot (**b**). Gapdh was blotted as a loading control. Representative gels are shown. **c**, IEC4.1-*Ifnar1*$^{-/-}$ cells do not respond to IFN-α stimulation. Cells were exposed to IFN-α (25 U/ml) for 2h followed by RT-qPCR analysis of selected type I IFN-controlled genes, including *Ifi44*, *Oas1g*, and *Usp18*, presented as fold changes to non-IFN-α treated control normalized to *Gapdh*. Data are from three biological replicates and presented as mean values ± SD (in a and c). *P* values were determined by two-tailed unpaired Student's *t*-test. Source data are provided as a Source Data file.

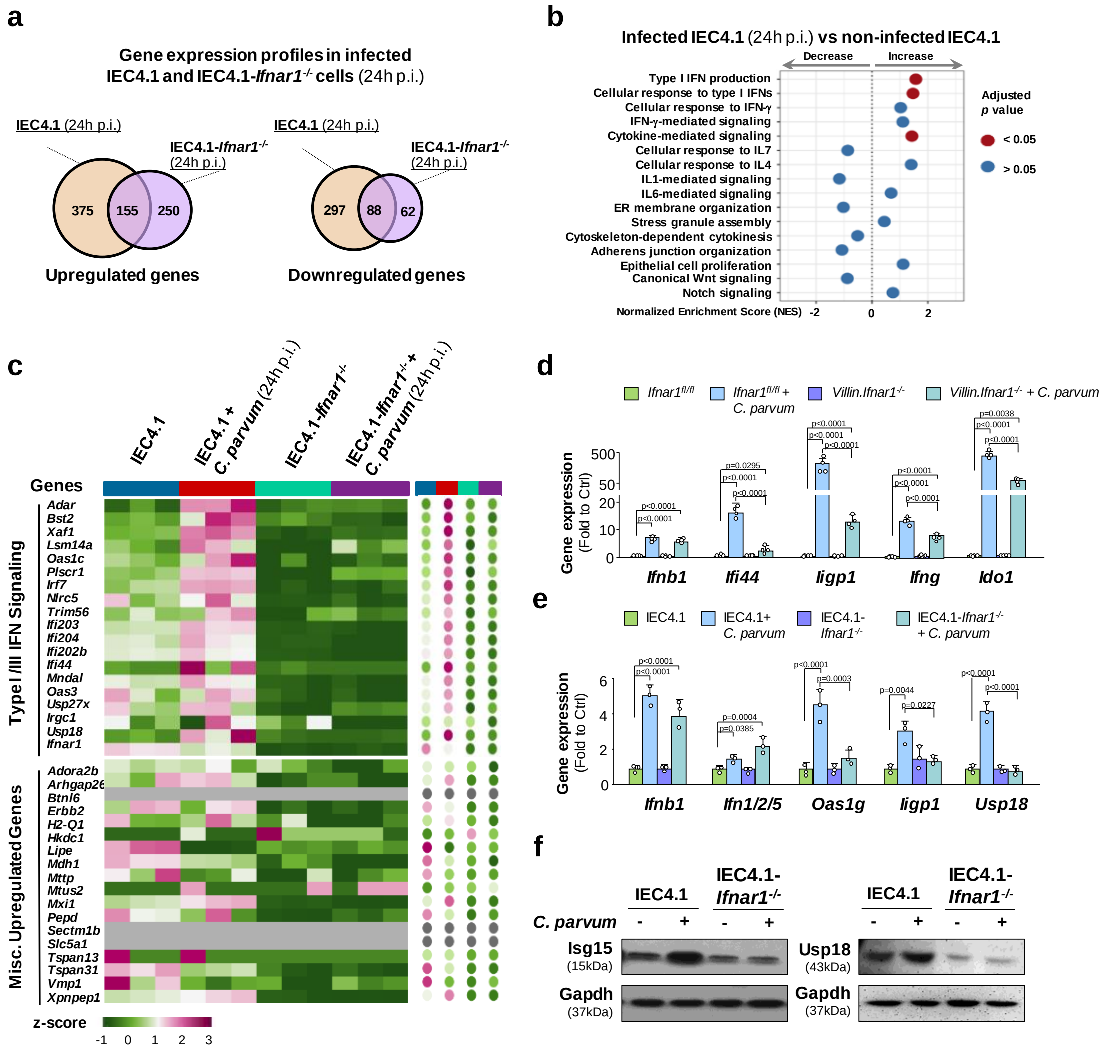

Supplementary Figure 3. Supplemental analysis about alterations in gene expression profiles, including various types of IFNs and IFN-stimulated genes, in neonates or IEC4.1 cell cultures following *C. parvum* infection

a, Gene expression profiles in IEC4.1 cell cultures with and without infection. IEC4.1 and IEC4.1-*Ifnar1*$^{-/-}$ cells were exposed to *C. parvum* infection for 24h, followed by RNA-Seq analysis. The number of genes whose expression levels were significantly upregulated or downregulated following infection was listed. Data are from three biological replicates (N=3 of RNA-seq replicates each group). **b**, Gene set enrichment analysis of gene expression profiles in the infected IEC4.1 cells (24h p.i.) compared with that in the uninfected control. Data are from three biological replicates (N=3 of RNA-seq replicates each group). *P* values were calculated based on Kolmogorov-Smirnov test and adjusted by Benjamini-Hochberg method. The normalized enrichment scores and adjusted p values for each function category are shown. **c**, Altered expression levels of selected gene groups from RNA-Seq analysis in IEC4.1 and IEC4.1-*Ifnar1*$^{-/-}$ cells following *C. parvum* infection (24h p.i.). Representative genes for the type I/III IFN signaling and misc. upregulated genes (whose expression levels were further increased in the infected *Villin.Ifnar1*$^{-/-}$ neonates) are shown. Data are from three biological replicates (N=3 of RNA-seq replicates each group). The heatmap represents expression levels for each replicate (N of 3 for each group) and the dots are the mean expression levels for each group. **d** and **e**, Expression levels of selected genes in intestinal epithelium from *Ifnar1*$^{fl/fl}$ and *Villin.Ifnar1*$^{-/-}$ neonates (**d**) and in IEC4.1 and IEC4.1-*Ifnar1*$^{-/-}$ cells (**e**) following *C. parvum* infection as measured by RT-qPCR. For in vivo infection, neonatal mice (5 days old) were orally inoculated with *C. parvum* oocysts (10^5 oocysts per animal) and ileal epithelium (2 cm of small intestine tissue from the ileocecal junction) was isolated (48h p.i.). For in vitro infection, IEC4.1 and IEC4.1-*Ifnar1*$^{-/-}$ cells were exposed to *C. parvum* infection for 24h. Expression levels of selected IFN genes were evaluated by RT-qPCR. Data were compiled from 3 independent experiments (**d**) or three biological replicates (**e**). *P* values were determined by two-way ANOVA followed by Tukey's HSD test (in **d** and **e**); $^{\#}$ $p < 0.05$, two-way ANOVA test compared with infected *Ifnar1*$^{fl/fl}$ animals (**d**) or infected IEC4.1 cells (**e**). **f**, Abundance of Isg15 and Usp18 proteins in IEC4.1 and IEC4.1-*Ifnar1*$^{-/-}$ cells following *C. parvum* infection (24h p.i.) by Western bot. Gapdh protein was blotted as a loading control and representative gels from three independent experiments are shown. Source data are provided as a Source Data file.

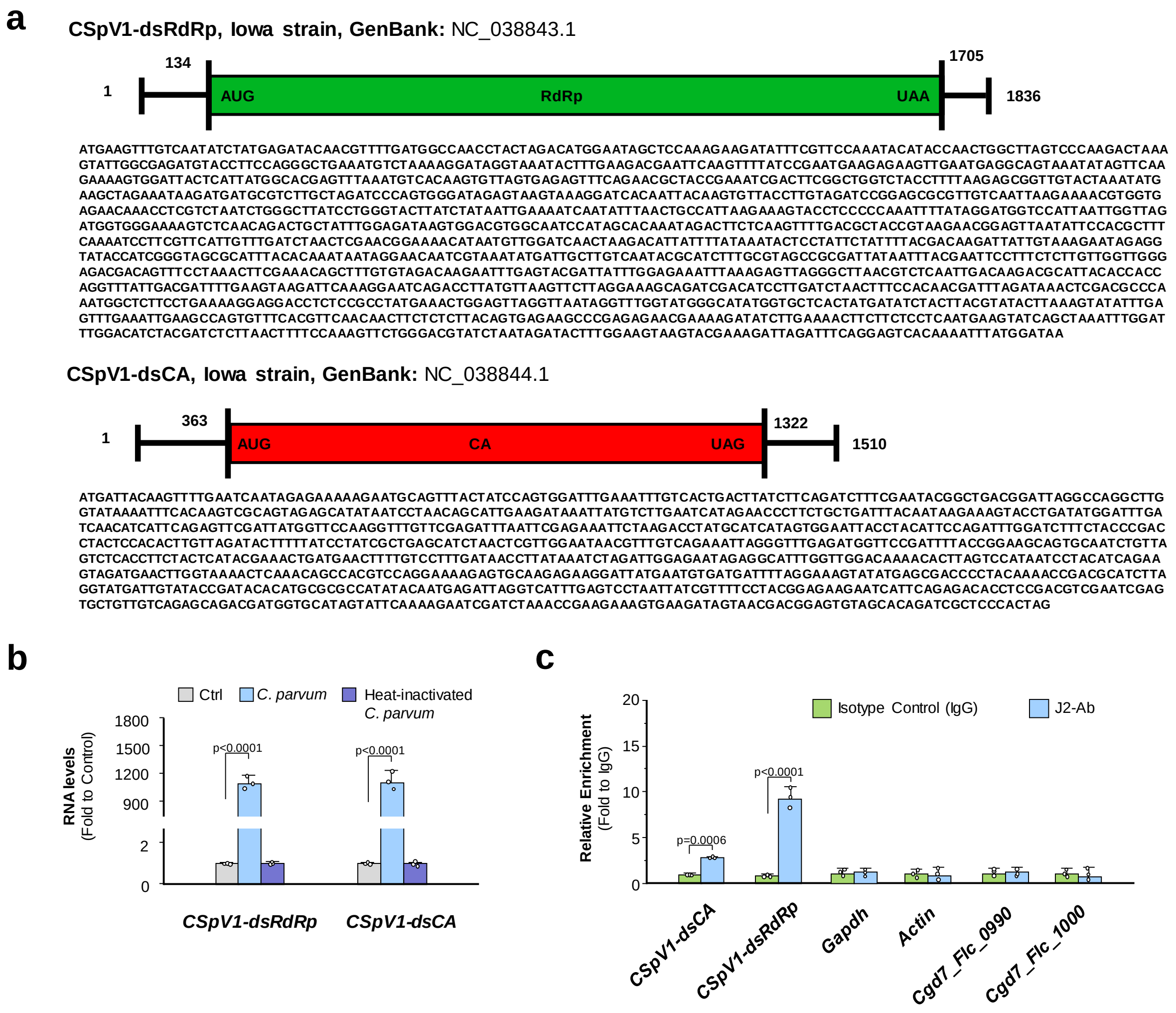

Supplementary Figure 4. Delivery of CSpV1-dsRNAs to infected host cells.
a, Schematic of the genome of CSpV1 comprising two distinct dsRNAs, *CSpV1-dsRdRp* and *CSpV1-dsCA*, and their full sequences. **b**, *CSpV1-dsRdRp* or *CSpV1-dsCA* was not detected in IEC4.1 cells after exposure to heat-inactivated *C. parvum*. Cells were exposed to *C. parvum* or heat-inactivated *C. parvum* (cultured in PBS at 65°C for 30 min) for 24h and cytoplasmic fractions were isolated and RNA levels were evaluated by RT-qPCR. *CSpV1-dsRdRp* and *CSpV1-dsCA* were measured by RT-qPCR. Data are presented as the fold change of the ratio to the host *Gapdh*. Cytoplasmic extract from cells collected from cell suspension mixed with the same amount of parasites was used as the control (Ctrl). **c**, Detection of selected RNA transcripts of cells origin and parasite origin by RIP assay using J2-antibody from infected IEC4.1 cells. Cells were exposed to *C. parvum* infection for 24h and cytoplasmic extracts were isolated for RNA immunoprecipitation using J2-antobody. Presence of CSpV1-RNAs and RNAs of IEC4.1 cells origin (*Gapdh* and *Actin*) and parasite origin (*Cgd7_FLc_0990* and *Cgd7_FLc_1000*) was measured by RT-qPCR. Data are from three biological replicates and presented as mean values ± SD. *p* values were determined by one-way ANOVA followed by Tukey's HSD test (in **b**) or by two-tailed unpaired Student's *t*-test (in **c**). Source data are provided as a Source Data file.

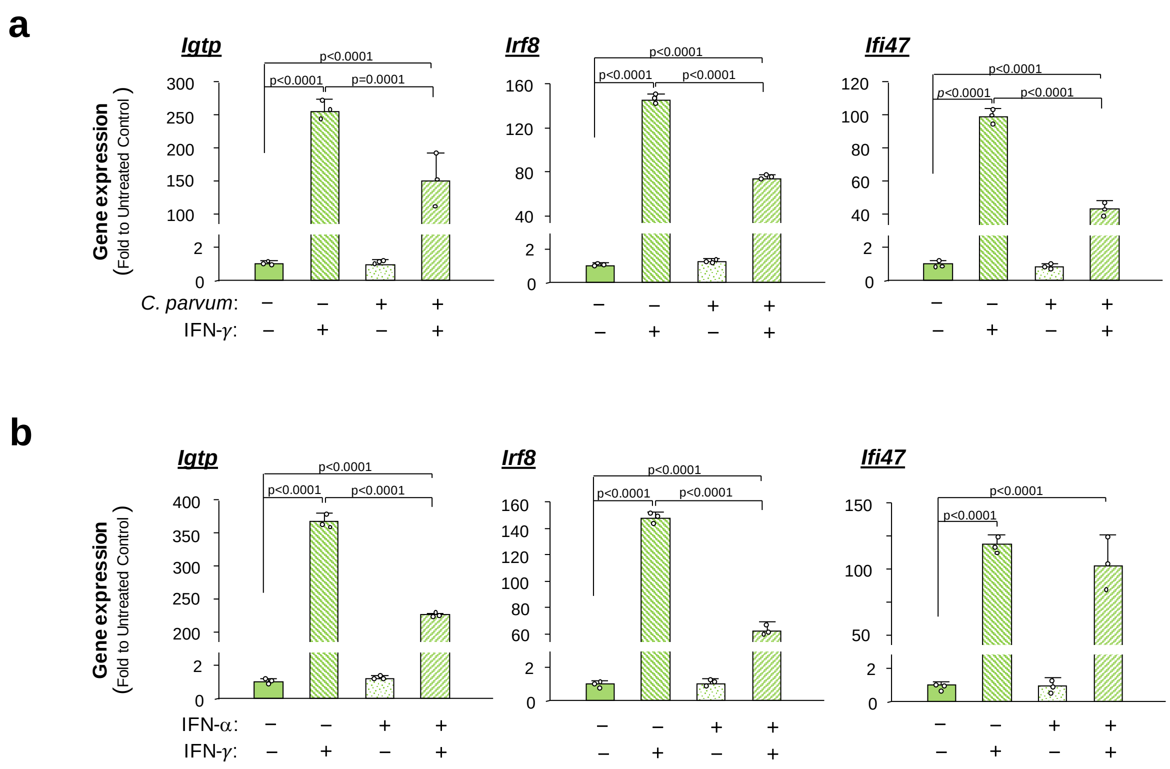

Supplementary Figure 5. Impaired cellular response to IFN-γ stimulation in *C. parvum*-infected IEC4.1 cells or cells pre-treated with IFN-α.

a and **b**, Impaired cellular response to IFN-γ stimulation in infected IEC4.1 cells (**a**) and cells pre-treated with IFN-α (**b**). IEC4.1 cells were first exposed to *C. parvum* infection for 24h followed by IFN-γ stimulation (1 ng/ml) for additional 2h (**a**) or first treated with IFN-α (25 U/ml) for 8h followed by IFN-γ stimulation for additional 2h in the absence of IFN-α (**b**). Expression levels of selected IFN-γ-stimulated genes (*Igtp, Irf8,* and *Ifi47*) were measured by RT-qPCR. Data are from three biological replicates and presented as mean values ± SD. *P* values were determined by two-way ANOVA followed by Tukey's HSD test. Source data are provided as a Source Data file.

Uncropped scans of blots and gels for the supplementary Figures.

Supplemental Fig. 1a

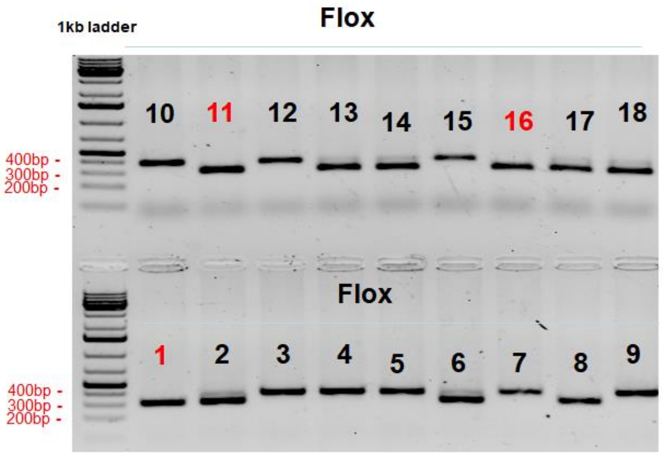

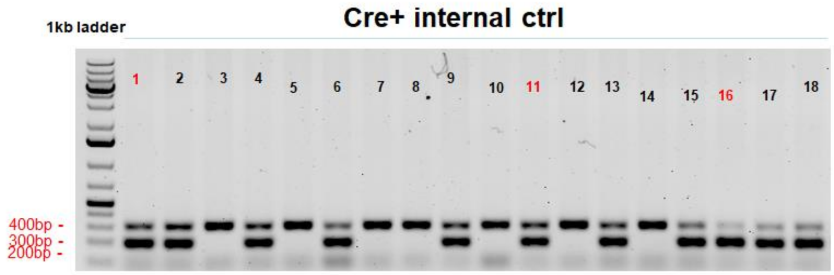

Supplemental Fig. 2b

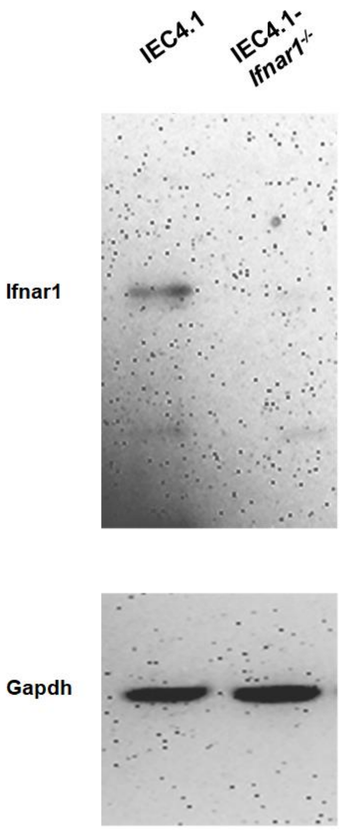

Supplemental Fig. 3f

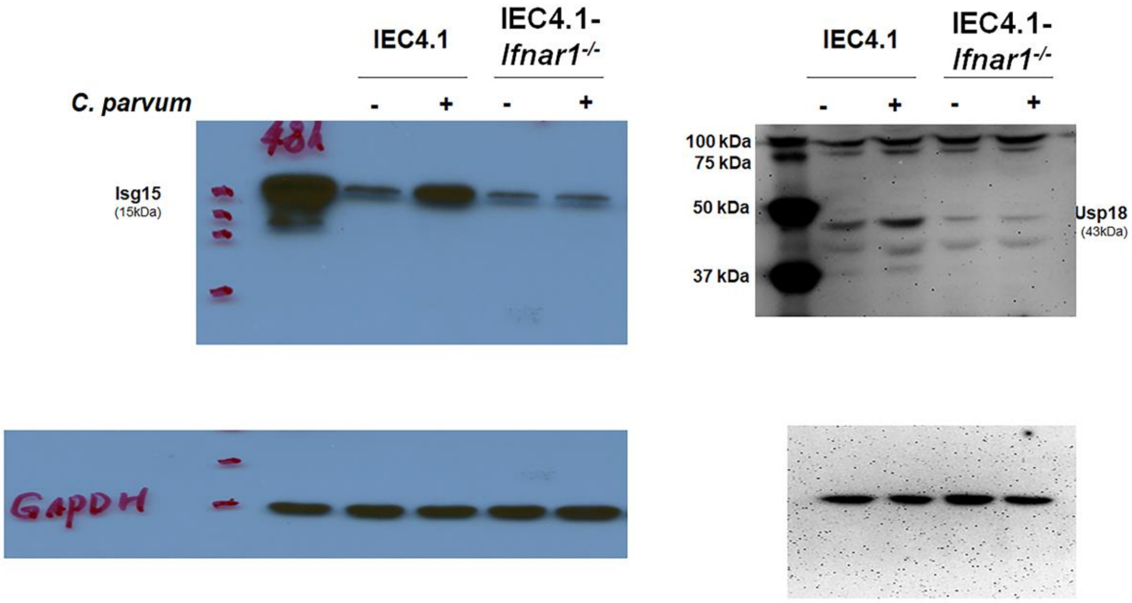

Supplement: Supplementary file 1 — Supplementary Information [file 41467_2023_37129_MOESM1_ESM.pdf]
