# Supplementary material for: Cryptosporidium uses CSpV1 to activate host type I interferon and attenuate antiparasitic defenses
Source: Nat Commun. 2023 Mar 16;14:1456. doi: 10.1038/s41467-023-37129-0 (PMC10020566; doi:10.1038/s41467-023-37129-0)
Supplement: Supplementary file 3 — Description of additional Supplementary Files [file 41467_2023_37129_MOESM3_ESM.pdf]

Description of Additional Supplementary Files

File name: Supplementary Data 1

Description: List of genes whose expression levels are significantly altered in the intestinal epithelium of infected neonates following *C. parvum* infection.

File name: Supplementary Data 2

Description: List of genes whose expression levels are significantly altered in IEC4.1 cells following *C. parvum* infection.

File name: Supplementary Data 3

Description: List of genes whose expression levels are significantly altered in IEC4.1 cells after transfection of CSpV1-dsRdRp or CSpV1-dsCA or in combination.

File name: Supplementary Data 4

Description: List of primers used for PCR and for generating constructs, siRNA sequences and probe sequences for *in situ* hybridization analysis.
