# Supplementary material for: Cryptosporidium uses CSpV1 to activate host type I interferon and attenuate antiparasitic defenses
Source: Nat Commun. 2023 Mar 16;14:1456. doi: 10.1038/s41467-023-37129-0 (PMC10020566; doi:10.1038/s41467-023-37129-0)
Supplement: Supplementary file 8 — Reporting Summary [file 41467_2023_37129_MOESM8_ESM.pdf]

nature portfolio

Corresponding author(s): Xian-Ming Chen

Last updated by author(s): 02-20-2023

Reporting Summary

Nature Portfolio wishes to improve the reproducibility of the work that we publish. This form provides structure for consistency and transparency in reporting. For further information on Nature Portfolio policies, see our Editorial Policies and the Editorial Policy Checklist.

Statistics

For all statistical analyses, confirm that the following items are present in the figure legend, table legend, main text, or Methods section.

n/a	Confirmed	
☐	☒	The exact sample size (*n*) for each experimental group/condition, given as a discrete number and unit of measurement
☐	☒	A statement on whether measurements were taken from distinct samples or whether the same sample was measured repeatedly
☐	☒	The statistical test(s) used AND whether they are one- or two-sided<br>*Only common tests should be described solely by name; describe more complex techniques in the Methods section.*
☒	☐	A description of all covariates tested
☐	☒	A description of any assumptions or corrections, such as tests of normality and adjustment for multiple comparisons
☐	☒	A full description of the statistical parameters including central tendency (e.g. means) or other basic estimates (e.g. regression coefficient) AND variation (e.g. standard deviation) or associated estimates of uncertainty (e.g. confidence intervals)
☐	☒	For null hypothesis testing, the test statistic (e.g. *F*, *t*, *r*) with confidence intervals, effect sizes, degrees of freedom and *P* value noted<br>*Give P values as exact values whenever suitable.*
☒	☐	For Bayesian analysis, information on the choice of priors and Markov chain Monte Carlo settings
☒	☐	For hierarchical and complex designs, identification of the appropriate level for tests and full reporting of outcomes
☒	☐	Estimates of effect sizes (e.g. Cohen's *d*, Pearson's *r*), indicating how they were calculated

Our web collection on statistics for biologists contains articles on many of the points above.

Software and code

Policy information about availability of computer code

Data collection	Bio-Rad CFX Manager v3.1 for qPCR<br>AlphaEase FC Software v6.0.2 for Western blot<br>BGI DNBSEQ for RNA sequencing<br>SoftMax Pro software v5.0.1 for ELISA
Data analysis	Image J v1.51 for image analysis<br>GraphPad prism 9.4.1 and MS Excel for statistical analysis<br>For RNA sequencing data analysis, programs: BGI SOAPnuke v1.5.2, HISAT v2.1.0, Bowtie2 v2.3.4.1<br>Dr. TOM data system for RNA sequencing data analysis.<br>R packages: clusterProfiler v4.4.4, ggplot2 v3.3.6, ComplexHeatmap v2.12.1.<br>Genomo alignment: Mus Musculus GRCm38 build 100 were used.<br>Venny 2.1 software for Venn diagrams

For manuscripts utilizing custom algorithms or software that are central to the research but not yet described in published literature, software must be made available to editors and reviewers. We strongly encourage code deposition in a community repository (e.g. GitHub). See the Nature Portfolio guidelines for submitting code & software for further information.

Data

Policy information about availability of data

All manuscripts must include a data availability statement. This statement should provide the following information, where applicable:

- Accession codes, unique identifiers, or web links for publicly available datasets
- A description of any restrictions on data availability
- For clinical datasets or third party data, please ensure that the statement adheres to our policy

Source data are provided with this paper.
RNA sequencing data generated in this study have been deposited to the NCBI GEO database under the accession number GSE147720 (https://www.ncbi.nlm.nih.gov/geo/query/acc.cgi?acc=GSE147720), GSE164279 (https://www.ncbi.nlm.nih.gov/geo/query/acc.cgi?acc=GSE164279), and GSE164316 (https://www.ncbi.nlm.nih.gov/geo/query/acc.cgi?acc=GSE164316).
Mus_musculus genome assembly (GCF_000001635.26_GRCm38.p6) is available in NCBI data bank. (https://www.ncbi.nlm.nih.gov/data-hub/genome/GCF_000001635.26/)

Human research participants

Policy information about studies involving human research participants and Sex and Gender in Research.

Reporting on sex and gender	N/A
Population characteristics	N/A
Recruitment	N/A
Ethics oversight	N/A

Note that full information on the approval of the study protocol must also be provided in the manuscript.

Field-specific reporting

Please select the one below that is the best fit for your research. If you are not sure, read the appropriate sections before making your selection.

☒ Life sciences ☐ Behavioural & social sciences ☐ Ecological, evolutionary & environmental sciences

For a reference copy of the document with all sections, see nature.com/documents/nr-reporting-summary-flat.pdf

Life sciences study design

All studies must disclose on these points even when the disclosure is negative.

Sample size	Sample size of all the experiments are described in figure legends, and the sample size were sufficient to conduct reasonable statistical analyses where applicable. Sample size was determined using power calculation allowing the use of statistical analysis. Statistical significance of differences between experimental groups and controls was assessed by two-tailed unpaired or paired t-test, and one or two way ANOVA, as indicated in the legends. In all cases, p values < 0.05 were considered as statistically significant.
Data exclusions	No data were excluded from the analysis.
Replication	All experiments were replicated independently. Number of repeats is provided in figure legends.
Randomization	Animals were randomly divided into experimental and control groups. Cells were plated at the same time and allocated and selected randomly for different treatments.
Blinding	The treatment of the mice was blinded to the investigator performing the parasite counting (Fig 1b). Considering the multiple aspects of the study using cell cultures, complete blinding of the investigators was not possible for the data collection. In fact, these experiments and data analysis were independently replicated by several Investigators in the team..

Reporting for specific materials, systems and methods

We require information from authors about some types of materials, experimental systems and methods used in many studies. Here, indicate whether each material, system or method listed is relevant to your study. If you are not sure if a list item applies to your research, read the appropriate section before selecting a response.

Materials & experimental systems

n/a	Involved in the study
☐	☒ Antibodies
☐	☒ Eukaryotic cell lines
☒	☐ Palaeontology and archaeology
☐	☒ Animals and other organisms
☒	☐ Clinical data
☒	☐ Dual use research of concern

Methods

n/a	Involved in the study
☒	☐ ChIP-seq
☒	☐ Flow cytometry
☒	☐ MRI-based neuroimaging

Antibodies

Antibodies used

1. Anti-mouse Isg15 (Santa Cruz, catalogue no. sc-166755, 1:100) for western blot.
2. Anti-mouse Usp18 (Biomatik, catalogue no. CAC08089, 1:500) for western blot.
3. Anti-mouse Ifngr1 (Thermo Fisher Scientific, catalogue no. MA5-35147, 1:500) for western blot.
4. Anti-mouse Mda5 (Abcam, catalogue no. Ab79055, 1:50) for western blot.
5. Anti-mouse Mda5 (ThermoFisher Scientific, catalogue no. 21775-1-AP,4 ug/ml) for RIP.
6. Anti-mouse Rig-1 (Proteintech, catalogue no. 20566-1-AP) for western blot (1:500) and RIP (4 ug/ml).
7. Anti-mouse Mavs (Proteintech, catalogue no. 14341-1-AP) for western blot (1:2000) and RIP (4 ug/ml).
8. Anti-mouse Pkr (Santa Cruz, catalogue no. sc-6282 AC, 1:200) for western blot.
9. Anti-mouse Pkr (Proteintech, catalogue no. 18244-1-AP, 4 ug/ml) for RIP.
10. Anti-viral dsRNA J2 (Millipore, catalogue no. MABE1134) for dot blot, IF (1:60), RIP (4 ug/ml).
11. Anti-mouse Gapdh (Santa Cruz, catalogue no. sc-32233, 1:1000)
12. Anti-DIG-Fluorescin (Sigma-Aldrich, catalogue no. 11 207 741 910, 1:200)
13. Anti-Mouse IgG Isotype Control (Cell Signaling, catalogue no. 5415S, 4 ug/ml) for RIP.
14. Anti-Rabbit IgG (Cell Signaling, catalogue no. 3900S, 4 ug/ml) for RIP.
15. Rabbit anti-C. parvum membrane protein serum (1:250).
16. Anti-PCNA (Cell Signaling, catalogue no. 13110, 1:1000) for IF.
17. Anti-EpCAM (Abcam, catalogue no. ab71916, 1:200) for IF.
18. Anti-Lysozyme (Abcam, catalogue no. ab108508, 1:500) for IF.
19. Anti-Villin (Santa Cruz, catalogue no. sc-58897, 1:200) for IF.

Secondary antibodies:

1. Anti-rabbit-HRP-conjugated (Santa Cruz, catalogue no. sc-2357, 1:5000)
2. Anti-mouse-HRP-conjugated (Santa Cruz, catalogue no. sc-516102, 1:5000)
3. Anti-mouse-Cruz fluor 488-conjugated (Santa Cruz, catalogue no. sc-516176, 1:200)
4. Anti-rabbit-Cruz fluor 488-conjugated (Santa Cruz, catalogue no. sc-516248, 1:200)
5. Anti-bovine Alexa fluor 594-conjugated (Jackson Immuno Research lab, catalogue no. 101-585-003, 1:250)

Validation

1. Anti-mouse Isg15 https://www.scbt.com/p/isg15-antibody-f-9
2. Anti-mouse Usp18 https://www.biomatik.com/antibodies/usp18-polyclonal-antibody-cat-cac08089/
3. Anti-mouse Ifngr1 https://www.thermofisher.com/antibody/product/IFNGR1-Antibody-clone-ARC0662-Recombinant-Monoclonal/MA5-35147
4. Anti-mouse Mda5 https://www.abcam.com/mda5-antibody-ab79055.html
5. Anti-mouse Mda5 https://www.thermofisher.com/antibody/product/IFIH1-Antibody-Polyclonal/21775-1-AP
6. Anti-mouse Rig-1 https://www.ptglab.com/products/DDX58-Antibody-20566-1-AP.htm
7. Anti-mouse Mavs https://www.ptglab.com/products/MAVS-Antibody-14341-1-AP.htm#product-information
8. Anti-mouse Pkr https://www.scbt.com/p/pkr-antibody-b-10
9. Anti-mouse Pkr https://www.ptglab.com/products/EIF2AK2-Antibody-18244-1-AP.htm
10. Anti-viral dsRNA J2 https://www.emdmillipore.com/US/en/product/Anti-dsRNA-Antibody-clone-rJ2,MM_NF-MABE1134-25UL
11. Anti-mouse Gapdh https://www.scbt.com/p/gapdh-antibody-6c5
12. Anti-DIG-Fluorescin https://www.sigmaaldrich.com/US/en/product/roche/11207741910
13. Anti-Mouse IgG Isotype Control https://www.cellsignal.com/products/primary-antibodies/mouse-g3a1-mab-igg1-isotype-control/5415
14. Anti-Rabbit IgG https://www.cellsignal.com/products/primary-antibodies/rabbit-da1e-mab-igg-xp-isotype-control/3900
15. Rabbit anti-C. parvum membrane protein serum: Zhu, G. et al. Mol Biochem Parasitol 134, 127–135 (2004).
16. Anti-PCNA https://www.cellsignal.com/products/primary-antibodies/pcna-d3h8p-xp-rabbit-mab/13110
17. Anti-EpCAM https://www.abcam.com/epcam-antibody-ab71916.html
18. Anti-Lysozyme https://www.abcam.com/lysozyme-antibody-epr29942-ab108508.html
19. Anti-Villin https://www.scbt.com/p/villin-antibody-1d2c3

Secondary antibodies:

1. Anti-rabbit-HRP-conjugated https://www.scbt.com/p/mouse-anti-rabbit-igg-hrp
2. Anti-mouse-HRP-conjugated https://www.scbt.com/p/m-igg-kappa-bp-hrp
3. Anti-mouse-Cruz fluor 488-conjugated https://www.scbt.com/p/mouse-igg-kappa-binding-protein-cruzfluor-488
4. Anti-rabbit-Cruz fluor 488-conjugated https://www.scbt.com/p/mouse-anti-rabbit-igg-cfl-488-mouse-anti-rabbit-igg-cfl-488
5. Anti-bovine Alexa fluor 594-conjugated https://www.jacksonimmuno.com/catalog/products/101-585-003

Eukaryotic cell lines

Policy information about cell lines and Sex and Gender in Research

Cell line source(s)

IEC4.1 cell line, Dr. Pingchang Yang (McMaster University, Hamilton, Canada).

Cell line source(s)	HCT-8 cell line, ATCC, catalogue no. CCL-244<br>L-Wnt-3A cell line, ATCC, catalogue no. CRL-2647
Authentication	HCT-8 cell line, ATCC, catalogue no. CCL-244, and L Wnt-3A cell line, ATCC, catalogue no. CRL-2647, are commercially available and we indicated the Research Resource identifier linking. The IEC4.1 cells were received from Dr. Pingchang Yang at the McMaster University and we indicated the resource information from the previous publications.
Mycoplasma contamination	Cell lines tested negative for mycoplasma.
Commonly misidentified lines<br>(See ICLAC register)	No commonly misidentified cell lines used in this study.

Animals and other research organisms

Policy information about studies involving animals; ARRIVE guidelines recommended for reporting animal research, and Sex and Gender in Research

Laboratory animals	Mouse strains used in this study:<br>1. Ifnar1 flox/flox, C57BL/6J Backgroud. (Jackson Laboratory, catalogue no. 028256)<br>2. Villin-Cre, C57BL/6J Backgroud. (Jackson Laboratory, catalogue no. 004586)<br>5-day-old mice were used for all in vivo experiments.
Wild animals	This study did not involve wild animals.
Reporting on sex	This study did not involve sex-based analysis.
Field-collected samples	This study did not involve field-collected samples.
Ethics oversight	All animal experiments were reviewed and approved by the Institutional Animal Care and Use Committees of the Creighton University School of Medicine at Omaha, Nebraska and were carried out in strict accordance with the recommendations in the Guide for the Care and Use of Laboratory Animals.

Note that full information on the approval of the study protocol must also be provided in the manuscript.
